# Cell-Free DNA Screening for Sex Chromosome Abnormalities and Pregnancy Outcomes, 2018–2020: A Retrospective Analysis

**DOI:** 10.3390/jpm12010048

**Published:** 2022-01-04

**Authors:** Yanmei Lu, Shihao Zhou, Siyuan Linpeng, Siyi Ding, Shihong Li, Yujiao Li, Liangcheng Shi, Jun He, Yalan Liu

**Affiliations:** 1 Department of Genetic Eugenics, Changsha Hospital for Maternal & Child Health Care Affiliated to Hunan Normal University, Changsha 410007, China; ztyandlym2016@hotmail.com (Y.L.); zhoushihao19771212@hotmail.com (S.Z.); linpengsiyuan@hotmail.com (S.L.); dsy15111325665@hotmail.com (S.D.); lishihong8017352@hotmail.com (S.L.); liyujiao8787@hotmail.com (Y.L.); shiliangcheng503574051@hotmail.com (L.S.); 2 Department of Otolaryngology Heard and Neck Surgery, Xiangya Hospital, Central South University, Changsha 410008, China

**Keywords:** non-invasive prenatal screening (NIPT), sex chromosome abnormality, sex chromosome aneuploidy, prenatal diagnosis, the termination of pregnancy (TOP)

## Abstract

To evaluate the efficacy of non-invasive prenatal screening (NIPT) for detecting fetal sex chromosome abnormalities, a total of 639 women carrying sex chromosome abnormalities were selected from 222,107 pregnant women who participated in free NIPT from April 2018 to December 2020. The clinical data, prenatal diagnosis results, and follow-up pregnancy outcomes of participants were collected. The positive predictive value (PPV) was used to analyze the performance of NIPT. Around 235 cases were confirmed with sex chromosome abnormalities, including 229 cases with sex chromosome aneuploidy (45, X (*n* = 37), 47, XXX (*n* = 37), 47, XXY (*n* = 110), 47, XYY (*n* = 42)) and 6 cases with structural abnormalities. The total incidence rate was 0.11% (235/222,107). The PPV of NIPT was 45.37% (235/518). NIPT accuracy for detecting sex chromosome polysomes was higher than that for sex chromosome monomers. The termination of pregnancy rate for fetal diagnosis of 45, X, and 47, XXY was higher than that of 47, XXX, and 47, XYY. The detection rate of fetal sex chromosome abnormalities was higher in 2018–2020 than in 2010–2012 (χ^2^ = 69.708, *P* < 2.2 × 10^−16^), indicating that NIPT is greatly efficient to detect fetal sex chromosome abnormalities.

## 1. Introduction

In 1997, Lo et al. discovered cell-free fetal DNA (cffDNA) in the peripheral blood of pregnant women, which led to the development of non-invasive prenatal screening (NIPT) [1]. NIPT has high sensitivity and specificity for the detection of trisomy 21 (T21), trisomy 18 (T18), and trisomy 13 (T13) [2,3,4] and was widely used in clinical settings in China and abroad in 2011 [5]. NIPT is used for the screening of sex chromosome aneuploidy since 2012 [6,7,8]. The detection of any abnormality is issued as an additional report. However, the accuracy and feasibility of NIPT for the screening of sex chromosome abnormalities are questionable [9,10]. We performed a retrospective and comprehensive analysis of 639 pregnant women carrying abnormal sex chromosomes who were selected from 222,107 local pregnant women who participated in free NIPT conducted by the financial expenditure of Changsha local governments from April 2018 to December 2020, and the following data was obtained: free non-invasive prenatal screening data, clinical data, prenatal diagnosis results, and follow-up pregnancy outcomes. We aimed to investigate the clinical effectiveness and practicability of NIPT in the screening of sex chromosomes through massively parallel sequencing using BGISEQ-500 and to provide data and information for clinical genetic counseling and prenatal diagnosis.

## 2. Materials and Methods

### 2.1. Subjects

In Changsha, 222,107 pregnant women participated in free NIPT from April 2018 to December 2020. A total of 639 cases of sex chromosome abnormalities were screened. Exclusion criteria included multiple pregnancies, absence of chromosomal abnormalities in any of the spouses, allogeneic blood transfusion, transplantation, allogeneic cell therapy, and immunotherapy within one year. All participants were provided with informed consent. The basic clinical features of the 639 pregnant women that were diagnosed with sex chromosome abnormalities by NIPT are presented in Table 1.

### 2.2. Methods

The age, height, weight, gestational week, the number of fetuses, adverse pregnancy history, mode of pregnancy, and serological screening results of pregnant women were obtained using the free livelihood project system and prenatal screening system. Pregnancy outcomes were obtained through follow-up by telephonic interviews, queries raised by pregnant women, and the prenatal care system and child health care system. The positive predictive value (PPV) was calculated as the number of NIPT cases that were concordant confirmatory diagnostic tests, divided by the number of cases with karyotype results of prenatal diagnosis (including mosaic cases), and multiplied by 100. R software (R 3.6.1 GUI 1.70 EI Capitan build (7684)) was used to perform the statistical analysis of the data. All countable data are presented in terms of frequency and rate. The chi-square test was performed to test statistical significance, and a *p*-value of <0.05 was considered statistically significant.

### 2.3. Experimental Methods

Peripheral blood (5 mL) was collected from pregnant women and stored in EDTA anticoagulant tubes (Kangwei Biological Technology Co., Ltd., Jiangsu, China) for no more than 96 h at 6–35 °C. The blood was centrifuged for 10 min at 1600× *g* and 16,000× *g* at 2 °C and 8 °C, respectively. The plasma was then collected in 2-mL nuclease-free centrifuge tubes for further use.

The cffDNA in the collected plasma was extracted for library construction (reagents were from BGI-Wuhan, Wuhan, China; the instrument for gene amplification was from Hangzhou Bo Technology Co., Ltd., Hangzhou, China). A combined prop-anchored polymorphic fetal chromosome aneuploidy detection kit (for T21, T18, and T13) (BGI-Wuhan, Wuhan, China) was used. Massively parallel sequencing was performed using BGISEQ-500 (Shenzhen Genomics Biomedical Engineering Co., Ltd., Shenzhen, China). Sequencing results were compared with the reference genomes (hg19, NCBI build 36), and z-scores were calculated for each chromosome. The results were interpreted using the software Halos-NIFTY (BGI-Shenzhen, Shenzhen, China). The above steps were performed according to the BGI instructions.

Pregnant women with abnormal sex chromosome results were informed to undergo genetic counseling. Amniocentesis or umbilical cord blood puncture was performed for fetal karyotyping and chromosomal microarray analysis using a CytoScan 750 K array (Affymetrix, Santa Clara, CA, USA).

**Figure 1:**
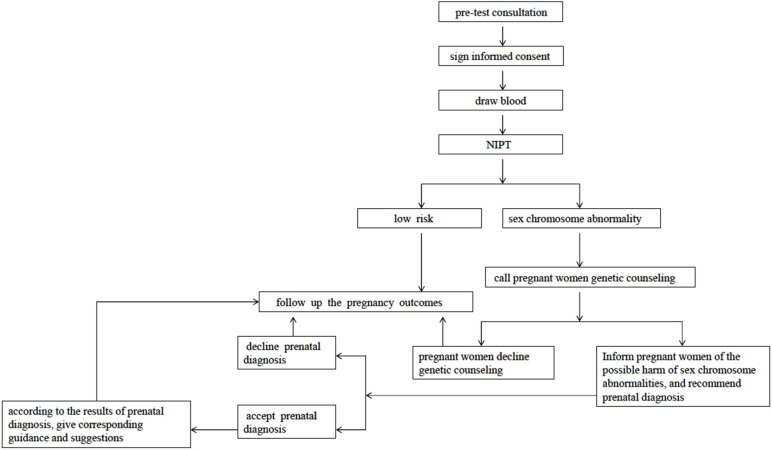
The detection of sex chromosome abnormalities by non-invasive prenatal screening.

The study protocol for the detection of sex chromosome abnormalities by NIPT is presented in Figure 1.

## 3. Results

### 3.1. NIPT, Prenatal Diagnosis Results, and Follow-Ups

Among 222,107 pregnant women who participated in free NIPT, 639 were detected with sex chromosome abnormalities. The positive rate of screening was 0.29% (639/222,107); the rate of prenatal diagnosis was 81.06% (518/639); the rate of follow-up was 99.22% (634/639); the total rate of termination of pregnancy (TOP) was 73.28% (170/232) (Table 2 and Table 3). After prenatal diagnosis, there were found unexpectedly karyotype results as follows: 46, XN, 9qh+; 46, XN, 21pss; 46, XN, inv (9) (p12q13); 46, XN, del (8) (q24.13–24.22); 47, XN, +21 (*n* = 2); and 47, XN, +18. The four abnormal karyotype fetuses with 46, XN, del (8) (q24.13–24.22); 47, XN, +21 (*n* = 2); and 47, XN, +18 underwent TOP. The risk of T21 and T18 in Down’s serological screening of T18 fetuses was high. The risk was low in other three cases with karyotype 46, XN, 9qh+; 46, XN, 21pss; 46, XN, inv (9) (p12q13). After follow-up, most pregnant women who refused to undergo prenatal diagnosis did not have their children’s peripheral blood karyotype analysis after full-term delivery. The peripheral blood karyotype was analyzed in only two cases, and the results were either normal or 47, XXY. Two pregnant women were diagnosed with 47, XXX karyotype; their NIPT results reported X (increased)-M, and their fetal karyotype results were normal. One pregnant woman had a karyotype of 45, X, and her NIPT result reported 45, X. However, she refused to undergo prenatal diagnosis and informed the situations of the fetus with 45, X after delivery. The NIPT results of another pregnant woman indicated 45, X karyotype. She directly underwent a TOP without prenatal diagnosis. Three pregnant women with 45, X karyotype underwent a prenatal diagnosis. Nuchal cystic hygroma was observed in their fetus during the ultrasonographic examination. One infant with a normal karyotype died due to pulmonary infection after birth.

### 3.2. Accuracy of NIPT in the Screening of Fetal Sex Chromosome Abnormalities

In this study, 235 cases of sex chromosome abnormalities were detected before delivery, which included 229 cases of abnormal number of chromosomes and 6 cases of structural abnormalities. The incidence rate was 0.11% (235/222,107), and the PPV was 45.37% (235/518) (Table 2 and Table 3).

### 3.3. Comparison between Increased and Decreased Aneuploidy Groups Detected by NIPT

NIPT results showed 307 cases of an increased chromosome aneuploidy, of which 189 cases were confirmed by chromosome karyotype with an accuracy rate of 61.56%, and 255 cases of a decreased chromosome aneuploidy, of which 37 cases were confirmed by chromosome karyotype with an accuracy rate of 14.51%. The difference between the two groups was statistically significant (χ^2^ = 56.888, *P* < 4. 61 × 10^−1^^4^).

### 3.4. Comparison between True-Positive and False-Positive Cases, Number of Births, and TOP in the Cases of Sex Chromosome Aneuploidy Detected by NIPT

Among the fetuses with sex chromosome aneuploidy detected by NIPT, 226 cases were true-positive, and 236 cases were false-positive. The difference between the two groups was statistically significant (*P* < 0.05). The cases included 384 newborn births and 167 TOPs, and the difference between the two groups was statistically significant (*P* < 0.05) (Table S1).

### 3.5. Pairwise Comparison between True-Positive and False-Positive Cases and between the Number of Births and TOP in the Cases of Sex Chromosome Aneuploidy Detected by NIPT

Significant differences in the number of true-positive cases and false-positive cases were observed in the following groups (*P* < 0.05): 45, X and 47, XXX; 45, X and 47, XXY; 45, X and 47, XYY; 47, and XXX and 47, XXY. (Table S2) Significant differences in the number of births and TOPs were observed in the following groups (*P* < 0.05): 45, X and 47, XXY; 47, XXX and 47, XXY; and 47, XXY and 47, XYY (Table S3).

### 3.6. Mosaic Karyotype of Sex Chromosome Abnormalities

Among the 639 pregnant women, 18 women were diagnosed with mosaic karyotype by prenatal diagnosis, with an incidence rate of 2.82% (18/639) (Table 4).

### 3.7. Changes in the Detection Rate of Sex Chromosome Abnormalities in Our Hospital after NIPT Was Performed

From 2010 to 2012, the average annual detection rate of fetuses with abnormal sex chromosomes by prenatal diagnosis in Changsha Maternity and Child Health Hospital was 0.76%, including 0.57% (3/524) in 2010, 0.69% (5/723) in 2011, and 0.94% (8/849) in 2012, because NIPT was not performed. After the use of NIPT from 2018 to 2020, the average annual detection rate of fetuses with abnormal sex chromosomes by prenatal diagnosis was 4.98%, including 5.65% (58/1027) in 2018, 4.61% (57/1236) in 2019, and 4.79% (62/1294) in 2020. The detection rate of fetal sex chromosome abnormalities in our hospital increased from 2018 to 2020 compared with that from 2010 to 2012, and the difference was statistically significant (χ^2^ = 69.708, *P* < 2. 2 × 10^−16^).

## 4. Discussion

### 4.1. Main Findings

The incidence rate of sex chromosome aneuploidy (SCAs) was 0.10%. The PPV of karyotypes 45, X; 47, XXY; 47, XXX; and 47, XYY was 18.14%, 58.73%, 80.29%, and 71.19%, respectively. The incidence rate of total PPV of SCAs was 44.21% (229/518). The PPV incidence rate of sex chromosome abnormalities was 45.37% (235/518). The results of our study are consistent with those of other related studies [11,12,13,14]. A significant difference was observed between the two groups that had an increase and decrease in the incidence of SCAs detected by NIPT. We observed that NIPT accuracy for sex chromosome trisomy was higher than that for sex chromosome monomers. This could be due to the lower guanosine-cytosine content of X chromosome and the age-related loss of X chromosome in male [15]. After prenatal diagnostic testing, the results were inconsistent with NIPT, which were probably due to the low fetal DNA fraction, maternal obesity, maternal copy number variations or mosaicism, abnormal maternal karyotype, confined placental mosaicism, a vanishing twin, and maternal neoplasm [16]. Among the inconsistent results, the following seven cases with other karyotype results were detected: 46, XN, 9qh+; 46, XN, 21pss; 46, XN, inv (9) (p12q13); 46, XN, del (8) (q24.13–24.22); 47, XN, +21 (*n* = 2); and 47, XN, +18. It showed that, even though NIPT indicated abnormality in the sex chromosome, there may be an autosomal abnormality. Based on the current NIPT guidelines, the ACMG proposes its use in an invasive procedure for positive cases [17], which was questioned by some pregnant women. Since the PPV of NIPT for sex chromosome abnormalities was low, couples can feel pressurized to undergo further prenatal diagnosis with sex chromosome abnormalities [6]. Abnormal sex chromosomes in pregnant women are an important reason for false-positive results of sex chromosome abnormality detection by NIPT [16]. Therefore, all NIPT cases should be examined by maternal peripheral blood karyotyping.

During the follow-up of pregnancy outcome, the TOP rate after prenatal diagnosis was 73.28% (170/232), which is different from that reported previously [18] but consistent with another report [19]. The TOP rate for fetal diagnosis of 45, X, and 47, XXY was higher than that of 47, XXX, and 47, XYY and consistent with other studies [15,20]. However, six cases with 45, X (including one non-mosaic type and five mosaic types), 23 cases with 47, XXX (including 22 non-mosaic types and one mosaic type), 12 cases with 47, XXY (including 11 non-mosaic types, one mosaic type, and one non-mosaic type diagnosed after birth), 29 cases with 47, XYY syndrome (including 27 non-mosaic types and two mosaic types), and one case with 46, X, del (X) (q22) were consistent with that of the mother. No obvious abnormality in the fetus was observed at birth. The clinical phenotype and severity of mosaic karyotype in the fetus after birth depend on the proportion of mosaic and normal cells [16]. The birth of fetuses with sex chromosome abnormalities may be affected by the information and guidance provided by the genetic counselor and age, education, economic status, pregnancy history, and acceptance by the parents.

### 4.2. Strengths and Limitations

Compared with other similar studies, our study has significance in clinical settings because of the use of a larger cohort and clinical data. The limitation is that we did not detect the peripheral blood karyotype and placental karyotype in pregnant women. Therefore, the identification of true-positive and false-positive NIPT results was impossible.

### 4.3. Interpretation

Sex chromosome abnormalities cause gonadal dysplasia and congenital malformation of genital mutilation, including abnormal sex chromosome numbers, abnormal sex chromosome structures, Y chromosome variations, and abnormal true and false hermaphroditism. SCAs are a common genetic disease characterized by an abnormal number of X or Y chromosomes, resulting in abnormal gene expression and sex hormone [21,22]. They include 45, X, 47, XXX, 47, XXY, 47, XYY, and their mosaic types [23]. SCA incidence in newborns is 1/400–1/500, which is higher than that the incidence of common trisomies, such as T21 (12.6/10,000), T18 (1.2–2.3/10,000), and T13 (1.4/10,000) [16,17,20]. Hence, the clinical presentation of SCAs should be identified. Turner syndrome (45, X) is the most common clinical SCA related to the complete or partial loss of an X chromosome, accounting for approximately 1 in every 2000–2500 female newborns. It is usually characterized by short stature, primary amenorrhea, lack of sex hormones, infertility, generally normal intelligence, and, more commonly, learning disabilities. Triple X syndrome (47, XXX) is related to an increase in the X chromosome, accounting for approximately 1/1000 female newborns, and is usually characterized by tall stature, motor and language defects, learning disabilities, and more. Klinefelter syndrome (47, XXY) is associated with the addition of an X chromosome, accounting for approximately 1 in every 450–1000 male newborns. It is characterized by a normal phenotype at birth, tall stature, small testicles in adolescence, infertility, irritable personality, violent tendency, high crime rate, and retarded intelligence [21]. Jacob syndrome (47, XYY) is caused by the non-separation of the paternal chromosome during meiosis II or post-zygotic mitosis, accounting for approximately 1/1000 male newborns. No obvious clinical abnormalities are observed apart from tall stature, accompanied by varying degrees of speech or language disorders, violent tendencies, behavioral problems, and neurocognitive disorders. Therefore, clinical diagnosis can be missed or delayed. Although trisomies are the most common chromosome aberration in SCAs because of the increase or loss of intact sex chromosomes, mosaic SCAs and abnormal sex chromosome structures also exist. 45, X is the only identified human monomer, and 47, XXX, 47, XXY, 47, XYY are SCA trisomies. Because the phenotypes are different, they are often insufficient for the diagnosis of trisomy SCAs. Only 50% of men with 47, XXY and 15% of men with 47, XYY are estimated to be clinically diagnosed [24]. The most commonly used prenatal screening methods are Down’s serological screening, ultrasonography, and NIPT. Down’s serological screening is mainly used to detect T21, T18, and neural tube defects. Only 165 pregnant women with high and medium risk by Down’s serological screening were present. Cases with 45, X and cervical cystoma were screened by ultrasonography and detection of T21, T18, T13, and neural tube defects. NIPT could detect T21, T18, T13, and sex chromosome abnormalities. Prenatal diagnosis is recommended for pregnant women with sex chromosome abnormalities to avoid the birth of children with serious birth defects and to decrease family, socio-economic, and emotional burden. For children that were born without prenatal diagnosis, genetic analysis should be performed soon to clarify the etiology of their condition. Targeted management and treatment are needed in childhood, adolescence, and adulthood to help them conceive naturally or through pre-implantation. The detection rate of fetal sex chromosome abnormalities increased significantly, from 0.76% (2010–2012) to 4.98% (2018–2020), which indicated that NIPT can effectively improve the detection rate of sex chromosome abnormalities. However, NIPT is a genome-wide and low-depth sequencing [25]. Therefore, to improve the PPV of sex chromosome abnormalities, the sequencing depth and gene coverage should be improved.

## 5. Conclusions

Although a certain false-positive rate for NIPT exists, it is effective for screening sex chromosome abnormalities. Genetic counseling before and after testing is important. All cases of sex chromosome abnormalities should be diagnosed by invasive prenatal diagnosis and karyotype examination of maternal peripheral blood for early diagnosis, decision, intervention, and treatment.

## Figures and Tables

**Table 1:** The basic clinical characteristics of pregnant women with 639 cases sex chromosome abnormalities for NIPT.

Charactristics	Cases (n)	Percentage (%)
**Maternal age (years old)**		
<35	566	88.58
≥35	73	11.42
**BMI (kg/m^2^)**		
<18.5	56	8.76
18.5–23.9	408	63.85
24–27.9	148	23.16
≥28	27	4.23
**Gestational age (Weeks)**		
9–13	29	4.54
13 ^+1^—14 ^+6^	19	2.97
15–20	544	85.13
≥20 ^+1^	47	7.36
**Number of fetus**		
Singleton	639	100
Twins	0	0
**History of adverse pregnancy and childbirth**		
Yes	146	22.85
No	493	77.15
**Method of conception**		
Natural conception	622	97.34
Assisted reproduction	17	2.66
**Mid term Down’s serological screening results**		
AFP-MoM		
<0.7	72	11.27
0.7–2.5	507	79.34
>2.5	4	0.63
HCG-MoM		
<0.5	48	7.51
0.5–2.0	437	68.39
>2.0	98	15.34
High risk (cut-off: T21 > 1/270, T18 > 1/270)	T21:59/T18:4	T21:9.23/T18:0.63
Medium risk (cut-off: T21:1/270-1/1000, T18:1/300-1/1000)	T21:87/T18:15	T21:13.62/T18:2.35
Low risk (cut-off: T21 < 1/270, T18 < 1/300)	T21:437/T18:564	T21:68.39/T18:88.26
Not performed	56	8.76

Weeks^+days.^

**Table 2:** Application of NIPT in the detection of fetal sex chromosome abnormalities and pregnancy outcomes.

Types of Sex Chromosome Abnormalities	NIPT (*n*)	Prenatal Diagnosis (*n*)		With Karyotype Analysis Results		PPV	Pregnancy Outcomes			
		Accepted (*n*)	Refused (*n*)	Accordance (*n*)	Discordance (*n*)		Followed-Up (*n*)	Births (*n*)	TOP (*n*)	Loss to Follow-Up (*n*)
45, X	255	204	46	37	167	18.14%	250	213	38	4
47, XXX	75	63	12	37	26	58.73%	75	58	17	0
47, XXY	158	137	21	110	26	80.29%	158	57	101	0
47, XYY	74	59	15	42	17	71.19%	74	63	11	0
X(increased)-M	45	33	12	2	31	6.06%	45	44	1	0
X(decreased)-M	28	19	9	0	19	0	28	27	1	0
Other complex for X	2	2	0	0	2	0	2	2	0	0
Del/Dup(X/Y)	2	1	1	1	1	50.00%	2	1	0	1
Total	639	518	116	229	289	44.21%	634	465	169	5

Abbreviation: IPT: non-invasive prenatal screening; PPV: positive predictive value; TOP: the termination of pregnancy.

**Table 3:** Analysis of fetal karyotype with abnormal sex X and Y chromosome variation.

Karyotype Results	NIPT	Maternal Age(Years Old)	Gestational Age (Weeks *)	BMI (kg/m^2^)	Number of Fetus	IVF (Yes/No)	History of Adverse Pregnancy and Childbirth	Abnormal Serological Screening Results	Follow Up
46, X, der(X) t(X; Y)(q23; q11.2)	XXY	27	19 ^+1^	23.74	Singleton	No	—	—	TOP
46, X,i(X)(q10)	XO	30	17 ^+5^	18.73	Singleton	No	—	Mid pregnancy: HCG-MoM:0.32	TOP
46, XYqh-(Y = 22)	XYY	31	19 ^+3^	25.91	Singleton	No	—	Mid pregnancy:HCG-MoM:3.00, T21:1/531	Male, no obvious abnormality in birth appearance
46, Xi(Y) (p10)	XO	28	13 ^+6^	17.78	Singleton	No	—	Mid pregnancy: HCG-MoM:0.43	TOP
46, X, del(X) q(21.3)	XO	32	17 ^+1^	22.89	Singleton	No	—	Mid pregnancy: HCG-MoM:0.27, T18:1/357	TOP
46, X, del(X) (q22)	del (Xq22.3-q28,46.57M)-M	32	16	25.65	Singleton	No	—	—	Female, no obvious abnormality in birth appearance, accordance with her mother’s karyotype of peripheral blood
									

NIPT, non-invasive prenatal screening; BMI, body mass index; IVF, in vitro fertilization; TOP, the termination of pregnancy. *, Weeks^+days.^

**Table 4:** Basic characteristics and follow-up of pregnant women with fetal mosaic chromosome karyotype results.

Mosaic Chromosome Karyotype Results	NIPT	Maternal Age (Years Old)	Gestational Age (Weeks *)	BMI (kg/m^2^)	Number of Fetus	IVF (Yes/No)	History of Adverse Pregnancy and Childbirth	Abnormal Serological Screening Results	Follow Up
45, X[26]/46, XX[54]	XO	26	19 ^+4^	26.23	Singleton	No	—	—	TOP
45, X[11]/46, XX[81]	XO	25	17 ^+3^	20.83	Singleton	No	—	PAPPA-MoM:3.39	TOP
47, XXX[18]/46, XX[37]	XO	28	16 ^+3^	17.04	Singleton	No	—	—	Female, no obvious abnormality in birth appearance
45, X[4]/46, XX[46]	XO	24	19	23.93	Singleton	No	—	second-trimester screening: HCG-MoM:2.31	Female, no obvious abnormality in birth appearance
45, X[15]/46, XX[86]	XO	32	12 ^+6^	26.35	Singleton	No	—	—	Female, no obvious abnormality in birth appearance
45, X[4]/46, XX[50]	XO	27	17 ^+2^	25.85	Singleton	No	—	—	Female, no obvious abnormality in birth appearance
45, X[8]/46, XX[92]	XO	29	18 ^+5^	27.27	Singleton	No	—	—	TOP, normal karyotype of peripheral blood with both husband and wife
47, XXX[33]/46, XX[62]	XXX	26	17 ^+4^	23.93	Singleton	No	—	—	Female, no obvious abnormality in birth appearance
45, X[30]/46, XY[70]	XO	40	17 ^+1^	24.14	Singleton	No	Spontaneous abortion three times	second-trimester screening: T21:1/400, T18:1/991	Male, no obvious abnormality in birth appearance
47, XYY[52]/46, XY[10]	XYY	30	16 ^+1^	26.45	Singleton	Yes	Spontaneous abortion once	PAPPA-MoM:2.18	Male, no obvious abnormality in birth appearance
45, X[15]/46, XX[65]	XO	25	16 ^+1^	19.72	Singleton	No	—	first-trimester screening: HCG-MoM:6.27, T21:1/61; second-trimester screening: HCG-MoM:5.36, T21:1/33	TOP
47, XYY[60]/46, XY[8]	XYY	23	17 ^+5^	20.7	Singleton	No	—	second-trimester screening: HCG-MoM:0.29	Male, no obvious abnormality in birth appearance
47, XXY[14]/46, XX[4]/46, XY[91]	XXY	37	17 ^+3^	20.34	Singleton	No	—	second-trimester screening: HCG-MoM:3.04, T21:1/80	Male, no obvious abnormality in birth appearance
48, XXXX[44]/47, XXX[6]	X(increased)-M	44	16 ^+2^	19.53	Singleton	No	—	second-trimester screening: AFP-MoM:2.37, T21:1/217, T18:1/812	Female, no obvious abnormality in birth appearance
45, X[19]/47, XXX[1]/46, XX[62]	XO	23	16 ^+6^	17.19	Singleton	No	—	second-trimester screening: HCG-MoM:5.7, T21:1/62	TOP
45, X[40]/46, XX[55]	XO	39	15 ^+5^	24.03	Singleton	No	Spontaneous abortion three times	first-trimester screening: T21:1/214; second-trimester screening: T21:1/145	TOP
45, X[50]/46, XY[50]	XO	31	12 ^+5^	22.67	Singleton	Yes	—	second-trimester screening: HCG-MoM:3.19, T21:1/65	TOP
45, X[6]/46, XX[94]	XO	37	19 ^+6^	22.03	Singleton	No	—	second-trimester screening: T21:1/205	Female, no obvious abnormality in birth appearance

NIPT, non-invasive prenatal screening; BMI, body mass index; IVF, in vitro fertilization; TOP, the termination of pregnancy. *, Weeks^+days^.

## Data Availability

Not applicable.

## Supplementary Materials

The following supporting information can be downloaded at: https://www.mdpi.com/article/10.3390/jpm12010048/s1, Table S1: Comparison between TP and FP, number of births and TOP in SCA cases. TP: true positive; FP: false positive; TOP: the termination of pregnancy; SCA: Sex chromosome aneuploidy. Table S2: Pairwise comparison of four types of SCA cases with TP and FP. SCA: Sex chromosome aneuploidy; TP: true positive; FP: false positive. Table S3: Pairwise comparison of four types of SCA cases with number of births and TOP. SCA: Sex chromosome aneuploidy; TOP: the termination of pregnancy.
