# Supplementary material for: Cell-Free DNA Screening for Sex Chromosome Abnormalities and Pregnancy Outcomes, 2018–2020: A Retrospective Analysis
Source: J Pers Med. 2022 Jan 4;12(1):48. doi: 10.3390/jpm12010048 (PMC8780735; doi:10.3390/jpm12010048)
Supplement: Supplementary file 1 [file jpm-12-00048-s001.zip › jpm-1497277-supplementary.pdf]

Table S1. Comparison between TP and FP, number of births and TOP in SCA cases.

	Cases with TP(n)	Cases with FP(n)	Number of births(n)	Number of TOP(n)
45，X	37	167	216	38
47, XXX	37	26	58	17
47, XXY	110	26	47	101
47, XYY	42	17	63	11
Total	226	236	384	167
χ^2	147.09		139.59	
P	<2.2E-16		<2.2E-16	

Abbreviation: TP: true positive ; FP : false positive ; TOP: The termination of pregnancy; SCA: Sex chromosome aneuploidy.

Table S2. Pairwise comparison of four types of SCA cases with TP and FP.

Number	Type	Cases with TP(n)	Cases with FP(n)	χ2	P
1	**45, X**	37	167	37.59	8.727E-10
	**47, XXX**	37	26		
2	**45, X**	37	167	128.35	< 2.2E-16
	**47, XXY**	110	26		
3	**45, X**	37	167	58.786	1.758E-14
	**47, XYY**	42	17		
4	**47, XXX**	37	26	9.8282	0.001719
	**47, XXY**	110	26		
5	**47, XXX**	37	26	1.5614	0.2115
	**47, XYY**	42	17		
6	**47, XXY**	110	26	1.7218	0.1895
	**47, XYY**	42	17		

Abbreviation: TP: true positive ; FP : false positive ; SCA: Sex chromosome aneuploidy.

Table S3. Pairwise comparison of four types of SCA cases with number of births and TOP.

Number	Type	Number of births(n)	Number of TOP(n)	χ^2	P
1	45, X	216	38	1.9472	0.1629
	47, XXX	58	17		
2	45, X	216	38	115.02	< 2.2E-16
	47, XXY	47	101		
3	45, X	216	38	2.3368E-31	1
	47, XYY	63	11		
4	47, XXX	58	17	39.69	9.76E-08
	47, XXY	47	101		
5	47, XXX	58	17	1.0184	0.3129
	47, XYY	63	11		
6	47, XXY	47	101	54.115	1.891E-13
	47, XYY	63	11		

Abbreviation: TOP: The termination of pregnancy.
